# Supplementary material for: Analysis of SARS-CoV-2 antibodies in COVID-19 convalescent blood using a coronavirus antigen microarray
Source: Nat Commun. 2021 Jan 4;12:6. doi: 10.1038/s41467-020-20095-2 (PMC7782488; doi:10.1038/s41467-020-20095-2)
Supplement: Supplementary file 1 — Supplementary Information [file 41467_2020_20095_MOESM1_ESM.pdf]

Supplementary Information

Supplementary Tables

Specimen Source	Specimen Type	Specimen Number	Symptom Days Median (Range)
University of California Irvine	Serum	45	11 (7-25)
University of California San Francisco	Plasma	63	11 (2-38)
University Hospital Basel	Plasma	13	33 (13-50)
Ortho Clinical Diagnostics	Serum	14	9 (7-22)
All Positive Specimens		135	11 (2-50)

Supplementary Table 1. SARS-CoV-2 PCR-positive convalescent blood specimens available for validation of the coronavirus antigen microarray. Each de-identified specimen was provided with associated data on symptom onset, positive PCR test, and collection.

Virus	Subtype	Strain	Protein	GenBank	Expression	Construct
Adeno	3	hAdV-3/45659	Fiber	P04501	E. coli	N-His-[Prot]-C
Adeno	3	hAdV-3/45659	Penton	Q2Y0H9	Baculovirus	N-His-[Prot]-C
Adeno	4	hAdV-4/28280	Fiber	P36844	Baculovirus	N-[Prot]-His-C
Adeno	4	hAdV-4/28280	Penton	Q2KSF3	Baculovirus	N-[Prot]-His-C
CoV	Alpha	229E	S1	A0A1L7B942	HEK293	N-(AA16-536)-His-C
CoV	Alpha	229E	S1+S2	A0A1L7B942	Baculovirus	N-(AA16-1115)-His-C
CoV	Alpha	NL63	S1	A0A1L2YVI8	HEK293	N-(AA19-717)-His-C
CoV	Alpha	NL63	S1+S2	A0A1L2YVI8	Baculovirus	N-(AA19-1296)-His-C
CoV	Beta	HKU1	HE	Q0ZME7	HEK293	N-(AA16-394)-His-C
CoV	Beta	HKU1	S1	YP_173238.1	HEK293	N-(AA1-760)-His-C
CoV	Beta	HKU1	S1	Q0ZME7	HEK293	N-(AA13-756)-His-C
CoV	Beta	HKU1	S1+S2	Q0ZME7	Baculovirus	N-(AA13-1295)-His-C
CoV	Beta	HKU23-368F	NP	AHN64796.1	HEK293	N-(AA1-448)-His-C
CoV	Beta	MERS	NP	AFS88943.1	Baculovirus	N-(AA1-413)-His-C
CoV	Beta	MERS	S1	AFS88936.1	HEK293	N-(AA1-725)-His-C
CoV	Beta	MERS	S1-RBD	AFS88936.1	Baculovirus	N-(AA383-502)-Fc-C
CoV	Beta	MERS	S1-RBD	AFS88936.1	Baculovirus	N-(AA367-606)-rFc-C
CoV	Beta	MERS	S2	AFS88936.1	Baculovirus	N-(AA726-1296)-His-C
CoV	Beta	OC43	HE	ATN39879.2	HEK293	N-(AA16-394)-His-C
CoV	Beta	OC43	S1	AVR40344.1	HEK293	N-(AA13-533)-His-C
CoV	Beta	OC43	S1+S2	AVR40344.1	Baculovirus	N-(AA13-1304)-His-C
CoV	Beta	SARS	NP	NP_828858.1	Baculovirus	N-(AA1-422)-His-C
CoV	Beta	SARS	PLpro	AAX16193.1	E. coli	N-(AA1541-1859)-His-C
CoV	Beta	SARS	S1	AAX16192.1	Baculovirus	N-(AA1-667)-His-C
CoV	Beta	SARS	S1-RBD	AAX16192.1	Baculovirus	N-(AA306-527)-His-C
CoV	Beta	SARS	S1-RBD	AAX16192.1	Baculovirus	N-(AA306-527)-Fc-C
**CoV**	**Beta**	**SARS-CoV-2**	**NP**		**Baculovirus**	**N-(AA)-His-C**
**CoV**	**Beta**	**SARS-CoV-2**	**PLpro**		**HEK293**	**N-(1564-1880)-His-C**
**CoV**	**Beta**	**SARS-CoV-2**	**S1**		**HEK293**	**N-(AA)-Fc-C**
**CoV**	**Beta**	**SARS-CoV-2**	**S1**		**Baculovirus**	**N-(AA)-His-C**
**CoV**	**Beta**	**SARS-CoV-2**	**S1**		**HEK293**	**N-(AA)-His-C**
**CoV**	**Beta**	**SARS-CoV-2**	**S1-RBD**		**HEK293**	**N-(AA)-mFc-C**
**CoV**	**Beta**	**SARS-CoV-2**	**S1-RBD**		**Baculovirus**	**N-(AA)-His-C**
**CoV**	**Beta**	**SARS-CoV-2**	**S1-RBD**		**HEK293**	**N-(AA)-His-C**
**CoV**	**Beta**	**SARS-CoV-2**	**S1-RBD**		**HEK293**	**N-(AA)-rFc-C**
**CoV**	**Beta**	**SARS-CoV-2**	**S1+S2**		**Baculovirus**	**N-(AA)-His-C**
**CoV**	**Beta**	**SARS-CoV-2**	**S2**		**Baculovirus**	**N-(AA)-His-C**
Flu	B	B/Malaysia/2506/2004	HA1	CO05957.1	HEK293	N-(AA1-362)-His-C
Flu	B	B/Malaysia/2506/2004	HA1+HA2	CO05957.1	HEK293	N-(AA1-556)-His-C
Flu	B	B/Phuket/3073/2013	HA1	EPI529345	HEK293	N-(AA1-361)-His-C
Flu	B	B/Phuket/3073/2013	HA1+HA2	EPI529345	Baculovirus	N-(AA1-547)-His-C
Flu	H1N1	A/Beijing/22808/2009	HA1	ADD64203.1	HEK293	N-(AA1-344)-His-C
Flu	H1N1	A/Beijing/22808/2009	HA1+HA2	ADD64203.1	HEK293	N-(AA1-529)-His-C
Flu	H3N2	A/Texas/50/2012	HA1	AGL07159.1	HEK293	N-(AA1-345)-His-C
Flu	H3N2	A/Texas/50/2012	HA1+HA2	AGL07159.1	Baculovirus	N-(AA1-530)-His-C
Flu	H5N1	A/Vietnam/1203/2004	HA1	AAW80717.1	HEK293	(AA1-342)-mFcg1-His
Flu	H5N1	A/Vietnam/1203/2004	HA1+HA2	AAW80717.1	HEK293	(AA1-531)-mFcg1-His
Flu	H7N9	A/Anhui/1/2013	HA1	AGJ51953.1	HEK293	N-(AA1-338)-His-C
Flu	H7N9	A/Anhui/1/2013	HA1+HA2	AGJ51953.1	HEK293	N-(AA1-524)-His-C
MPV	A	PER/CFI0320/2010/A	G		HEK293	N-(AA52-228)-His-C
MPV	B	PER/CFI0320/2010/A	F		HEK293	N-(AA280-490)-His-C
MPV	B	PER/CFI0466/2010/B	G		HEK293	N-(AA52-238)-His-C
PIV	1	12O3	F	A0A1V0E1X5	Baculovirus	N-(AA22-497)-His-C
PIV	1	12O3	H	A0A1B2CW87	Baculovirus	N-His-(AA60-575)-C
PIV	3	USA/10991B/2010	H	T1UD13	Baculovirus	N-His-(AA55-575)-C
PIV	4	hPIV-4b/10-H2/2016	H	A0A1V0E1N4	Baculovirus	N-His-(AA48-575)-C
RSV	A	LA2-94/2013	F	A0A023RA53	Baculovirus	N-(AA1-526)-His-C
RSV	A	LA2-94/2013	G	A0A076FRQ0	HEK293	N-(AA64-321)-His-C
RSV	B	B1	G		HEK293	N-(AA67-299)-His-C
RSV	B	TH-10526/2014	F	K7WLI9	Baculovirus	N-(AA1-525)-His-C

Supplementary Table 2. Content of coronavirus antigen microarray. The virus group, subtype, and strain, protein, GenBank identification where available, expression system, and gene construct are shown for each antigen, with the SARS-CoV-2 antigens indicated in bold. All antigens are sourced from Sino Biological Inc. in Beijing, China.

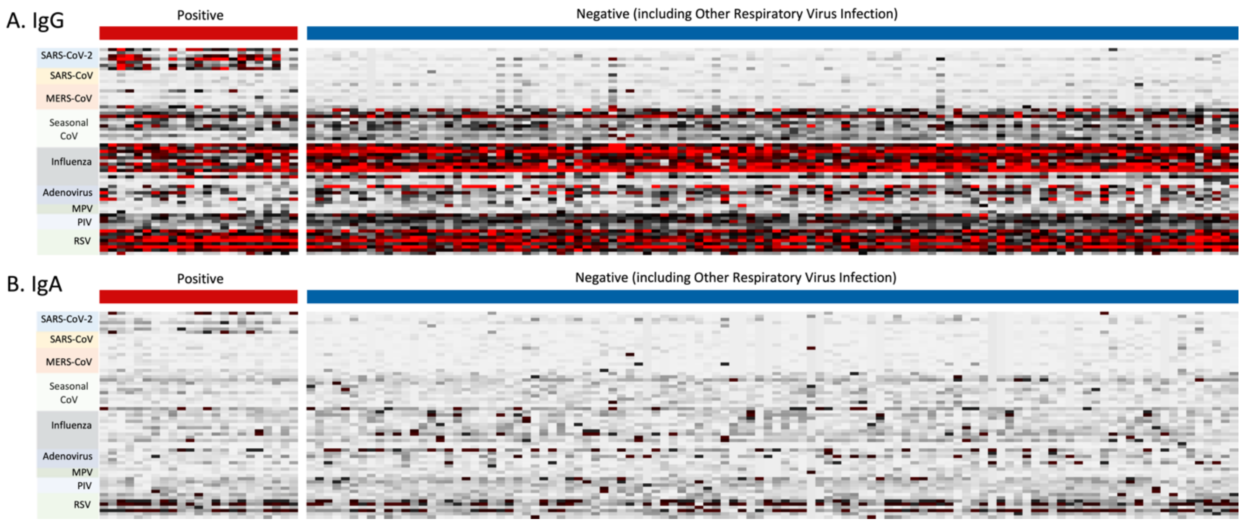

Supplementary Figure 1. Heatmap for coronavirus antigen microarray tested against college dormitory cohort monitored for respiratory virus infections. The heatmap shows IgG (A) and IgA (B) reactivity measured as mean fluorescence intensity across four replicates, against each antigen organized into rows color coded by virus, for blood specimens organized into columns classified as positive (convalescent from PCR-positive individuals) or negative (prior to pandemic from naïve individuals monitored for other respiratory virus infections). Reactivity is represented by color (white = low, black = mid, red = high).

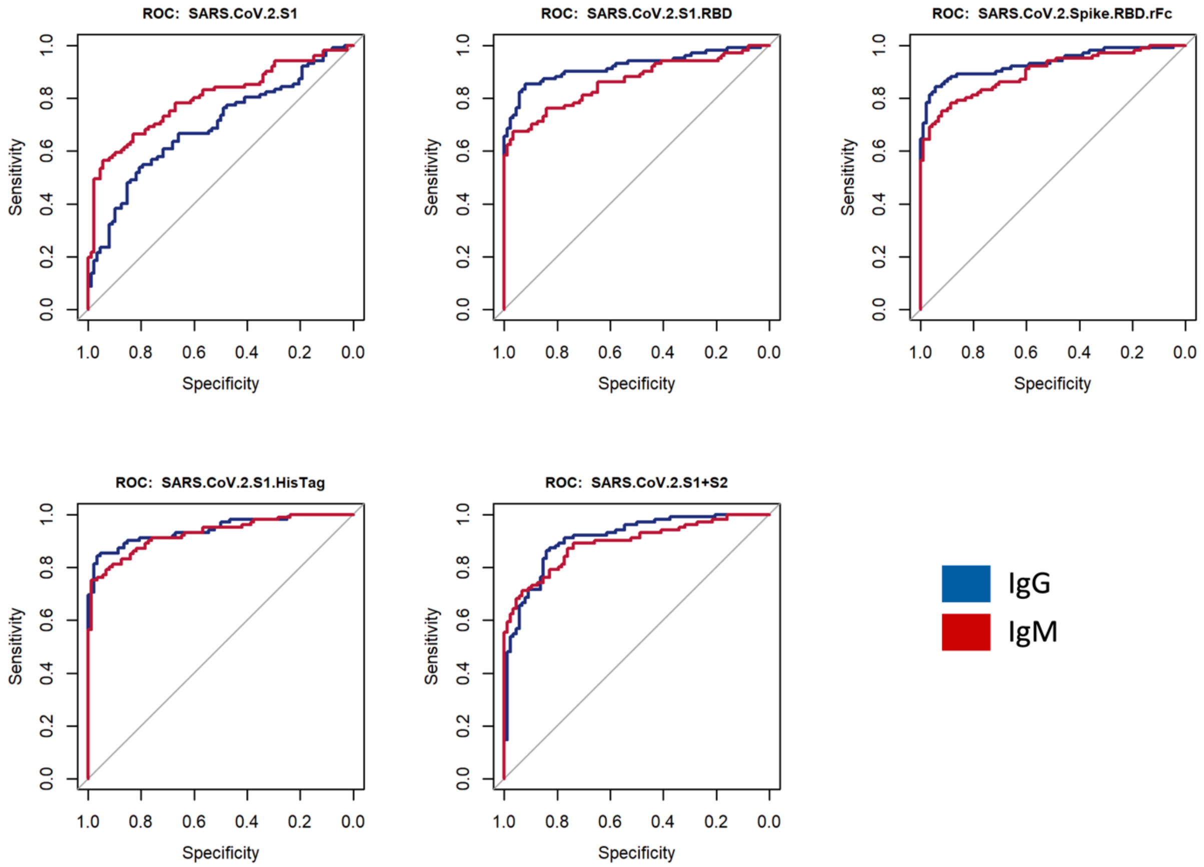

Supplementary Figure 2. ROC curves for high-performing antigens. ROC curves showing sensitivity versus specificity for discrimination of positive and negative individuals were derived for each individual high performing antigen (ROC AUC $\geq$ 0.95) for both IgG (blue line) and IgM (red line) and compared to no discrimination (ROC AUC = 0.5, dashed black line).

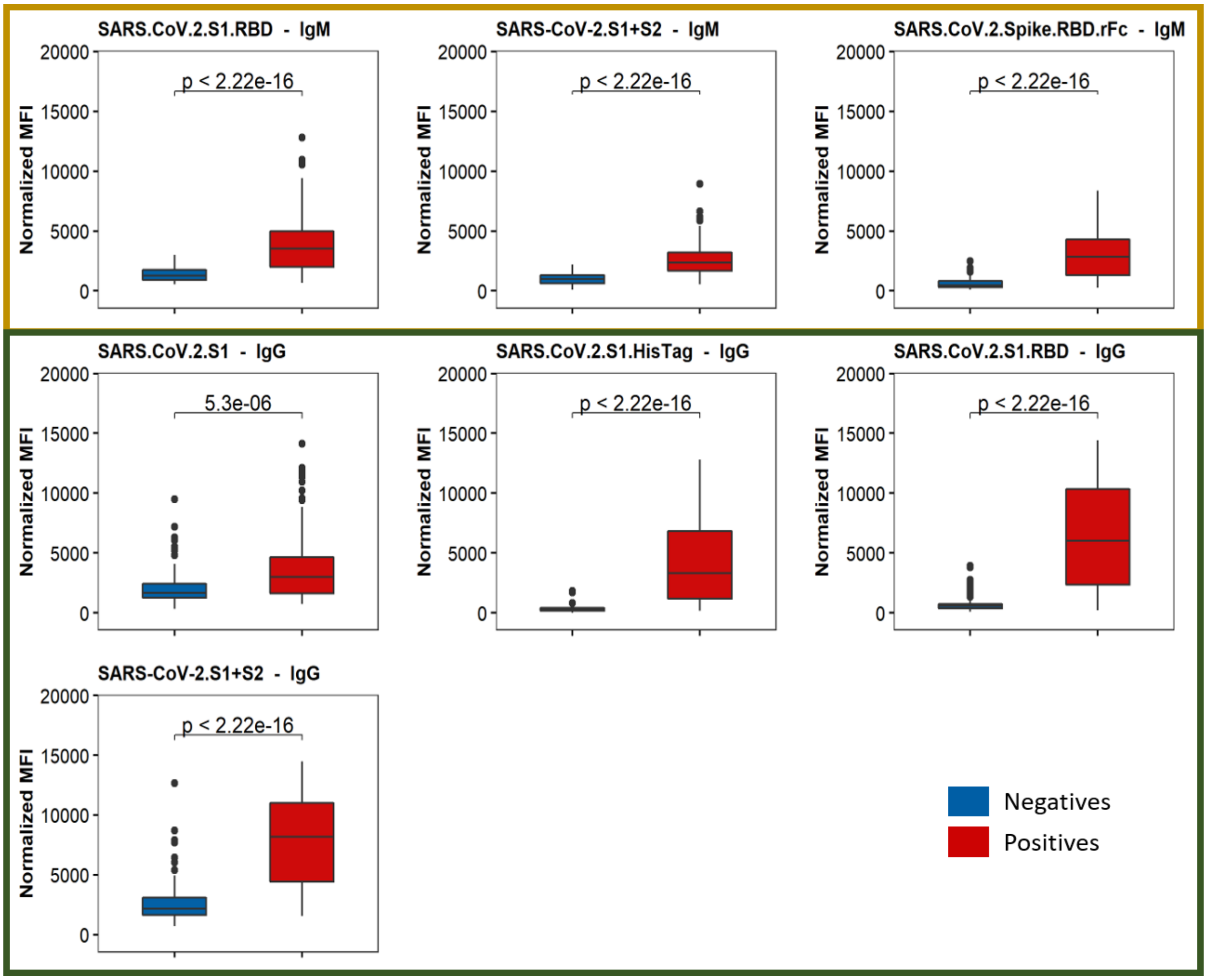

Supplementary Figure 3. Normalized antibody reactivity of positive and negative sera for high-performing antigens. IgG and IgM reactivity against each high-performing antigens (ROC AUC ≥ 0.95) measured as mean fluorescence intensity (MFI) for convalescent blood specimens from PCR-positive individuals (positive, red, n = 100 biologically independent samples) and from naïve individuals prior to pandemic (negative, blue, n = 88 biologically independent samples) are shown. The boxes represent the first quartile, median and third quartile and the wiskers extend 1.5 times the interquartile range (IQR) and dots represent individual sera outliers. Statistical analysis was done using the two-sided, unpaired, Wilcoxon test and $p < 0.05$ considered significant.
