# Supplementary material for: Analysis of SARS-CoV-2 antibodies in COVID-19 convalescent blood using a coronavirus antigen microarray
Source: Nat Commun. 2021 Jan 4;12:6. doi: 10.1038/s41467-020-20095-2 (PMC7782488; doi:10.1038/s41467-020-20095-2)
Supplement: Supplementary file 2 — Description of Additional Supplementary Files [file 41467_2020_20095_MOESM2_ESM.pdf]

Description of Additional Supplementary Files

File: Supplementary Data 1

Description: Diagnostic performance of all SARS-CoV-2 antigen combinations in discriminating positive and negative controls
